# Comparative Assessment of the Effects of Hydroxyethyl Starch and Normal Saline on Severe Hypotension in Patients with Aluminum Phosphide Poisoning: A Retrospective Study

**DOI:** 10.1155/2022/4985120

**Published:** 2022-03-09

**Authors:** Amin Nakhostin-Ansari, Gholamabbas Kafi, Mohammad Arefi, Nasrin Barzegari Dahaj, Samaneh Akbarpour, Asieh Mansouri, Behnam Behnoush, Davood Soroosh

**Affiliations:** ^1^Sports Medicine Research Center, Neuroscience Institute, Tehran University of Medical Sciences, Tehran, Iran; ^2^Forensic Medicine and Toxicology, Tehran University of Medical Sciences, Tehran, Iran; ^3^Baharloo Hospital, School of Medicine, Tehran University of Medical Sciences, Tehran, Iran; ^4^Baharloo Hospital, Tehran University of Medical Sciences, Tehran, Iran; ^5^Department of Epidemiology and Biostatistics, School of Public Health, Tehran University of Medical Sciences, Tehran, Iran; ^6^Occupational Sleep Research Center, Baharloo Hospital, Tehran University of Medical Sciences, Tehran, Iran; ^7^Hypertension Research Center, Cardiovascular Research Institute, Isfahan University of Medical Sciences, Isfahan, Iran; ^8^Department of Forensic Medicine, Sabzevar University of Medical Sciences, Sabzevar, Iran

## Abstract

**Background:**

Aluminum phosphide poisoning is one of the most common forms of poisoning which requires immediate and urgent treatment.

**Objective:**

This study aimed to compare the efficiency of two solutions, including hydroxyethyl starch and normal saline, in treating hypotension in patients with aluminum phosphide poisoning.

**Methods:**

This retrospective cohort study was conducted on 35 patients with aluminum phosphide poisoning. We reviewed the profile of 18 patients treated with hydroxyethyl starch and 17 patients treated with normal saline. Within-group and between-group differences in systolic blood pressure before and after treatment were compared using paired *t*-test and independent *t*-test, respectively.

**Results:**

The mean ± standard deviation (SD) age of the subjects in the starch and normal saline groups was 27.06 ± 9.72 and 27.88 ± 9.08, respectively. The levels of blood pressure in the two groups were not significantly different before the treatment; the mean ± SD of systolic blood pressure in the starch and normal saline groups was 72.67 ± 14.49 and 68.59 ± 8.3, respectively (*P*=0.313). After the treatment, it was significantly increased to 94 ± 24.45 and 85.18 ± 19.9 in the starch group (*P*=0.001) and the normal saline group (*P*=0.004), respectively. However, there was no significant difference between the two groups (*P*=0.245). Only one person survived in each group.

**Conclusion:**

According to the results of this study, although there was no significant difference between the two groups in terms of their effects on hypotension, these treatments could not prevent mortality.

## 1. Introduction

Rice tablet, with the scientific name of aluminum phosphide, is a chemical compound commonly used as an active rodenticide and insecticide to protect cereals [1]. The rice tablet available in Iran, with the commercial name of Phostoxin, contains aluminum phosphide as the main active ingredient; in addition, about 55% of the tablet is composed of urea, carbamate, and ammonium. The tablets are packed in cylindrical packs; each contained ten three-gram tablets with a dark gray color, and the package is sold at a low price [2–4].

Intentional poisoning using a rice tablet, as a type of suicide, is very common in Iran. Accordingly, one of the most common types of poisonings in Iran and India is the poisoning caused by rice tablets [5]. In particular, in the agricultural season, there are usually many deliberate cases of poisoning caused by rice tablets. Usually, each rice tablet contains three grams of aluminum phosphide, and taking even half of the tablet could be lethal [1]. After absorption in the liver, aluminum phosphide is metabolized, and its phosphine gas is slowly released, which can cause a delay in the emergence of the symptoms of intoxication. Early symptoms include chest pain, epigastric pain, vomiting, and low blood pressure [5]. These symptoms start quickly after 30 minutes, and in severe poisoning, death can occur in less than an hour [6]. The factors that seem to play a role in patients' prognosis include repeated vomiting and severe shock after using the drug. The most important factor is the patient's response to the treatments for severe hypotension; in order to treat the patient, it is necessary to restore the venous fluids lost due to the release of fluid into the third space. Previous studies have shown that although it is highly expected to observe an increase in central venous pressure due to left ventricular hypokinesis, high-dose and high-volume venous fluid administration does not cause severe impairment in these patients. Therefore, it is necessary to preserve systolic blood pressure above 80 mmHg by administering dopamine and dobutamine [4].

So far, a limited number of studies have been conducted on aluminum phosphide poisoning, and they have often been conducted as descriptive studies; only a few studies have focused on the treatment of patients [7, 8].

The administration of hydroxyethyl starch has had promising effects in the improvement of renal function in patients with severe burns and sepsis [9, 10]; thus, this study aimed to evaluate the effect of this substance on the correction of severe hypotension in patients with aluminum phosphide poisoning.

## 2. Methods

This study was conducted as a retrospective cohort study. All procedures performed in the study were in accordance with the ethical standards of the national research committee and with the 1964 Helsinki Declaration and its later amendments or comparable ethical standards. The Ethics Committee of Tehran University of Medical Sciences approved this study under approval number IR.TUMS.VCR.REC.1395.1741.

The study population included all the patients admitted to the poisoning intensive care unit (ICU) in Baharloo Hospital for four years, from April 2012 to April 2015. Based on their history and clinical symptoms, patients who were diagnosed with aluminum phosphide poisoning, had experienced metabolic acidosis (bicarbonate less than 14) during the first six hours, and had systolic blood pressure below 90 mmHg were enrolled into the study. Since starch is routinely administered in hospitals based on physicians' diagnoses, we reviewed the profile of patients undergoing starch treatment and compared them with those who had received normal saline in the hospital. A questionnaire was designed to collect the patients' demographic data (including age and sex) and the data on the disease, including systolic blood pressure in the first 24 hours of admission, duration of administration before admission to the ICU, length of hospitalization in the ICU, and the death of the patient.

There were variations in the fluid administration protocol based on the patients' conditions. However, 500 cc of the fluid were initially administrated for patients in one hour. After that, the patients received 1000 cc of the fluid in 4 to 6 hours. We read the systolic blood pressures that were recorded before starting the fluid administration and 30 to 60 minutes after the end of fluid administration.

SPSS16 software was used to analyze the data. The continuous and categorical variables were described by mean ± standard deviation (SD) and number (%). Since the sample size was not very large, the nonparametric tests were used for nonnormal items after conducting the normality test. A paired *t*-test (and in the case of nonnormalization, the Wilcoxon test) was used to compare the items before and after the intervention; in addition, an independent *t*-test (and in the case of nonnormalization, its equivalent nonparametric test, i.e., Mann–Whitney test) was used to compare the differences between the two groups. Categorical variables were compared between the two groups by the chi-squared test.

## 3. Results

In the present study, a total of 35 poisoned people were enrolled, 18 (51.4%) of whom were in the starch treatment group and 17 (48.6%) in the normal saline group. The mean ± SD of age in the starch and normal saline groups were 27.06 ± 9.72 and 27.88 ± 9.08, respectively. The range of age in all participants was between 14 and 47 years. The demographic characteristics of the participants are presented in Table 1. As shown, most of the participants were in the age of 20–30 years, male, and self-employed and had a diploma degree.

The time from taking the tablets until hospitalization was nonsignificantly (*P*=0.303) longer in the starch group compared with the normal saline group (11.3 hours ± 22.18 versus 3.14 hours ± 1.91, respectively).  Table 2 presents the results of comparing differences in systolic blood pressure within and between study groups. As shown, the blood pressure of the subjects in the starch group at the admission was 72.67 mmHg (SD = 14.49), on average, which significantly changed to 94 mmHg (SD = 24.45) after the treatment. In other words, their blood pressure significantly increased by approximately 21.33 mmHg (SD = 19). In the normal saline group, the mean systolic blood pressure significantly increased from 68.59 mmHg (SD = 8.3) before the treatment to 85.18 mmHg (SD = 22.8) after the treatment. In other words, their blood pressure significantly increased by approximately 16.58 mmHg (SD = 18.75). A comparison of the before and after the treatment differences between the two groups showed no statistically significant difference (*P*=0.463). It is worth mentioning that there was no significant difference between the two groups in terms of systolic blood pressure at the admission time (*P*=0.313).

17 persons (94.4%) in the starch group and 16 persons (94.1%) in the normal saline group died. However, there was no significant difference between groups regarding this treatment outcome (*P*=1). The length of hospitalization was not significantly different (*P*=0.106) in the normal saline group (mean = 17.53 hours, SD = 13.46) compared to the starch group (mean = 26.36 hours, SD = 17.89).

## 4. Discussion

This study evaluated the effect of hydroxyethyl starch, as compared with normal saline, in the treatment of severe hypotension in patients with aluminum phosphide poisoning via a retrospective cohort design. The results of the study showed that both normal saline and hydroxyethyl starch can increase the blood pressure of the subjects, and there was a statistically significant change before and after the treatment in each group. However, there was no significant difference between the two groups in terms of the level of changes. In fact, although starch was approximately more effective than normal saline in increasing the blood pressure after the treatment, the difference between the two groups was not statistically significant.

In a study in 2011, Marashi et al. proposed a hypothesis stating that hydroxyethyl starch, despite the controversies about its effects and its complications, could be a good candidate for the treatment of rice tablet poisoning and prevent hypotension [5].

After presenting this hypothesis, a case report in 2014 showed that the administration of hydroxyethyl starch could control acidosis in a patient who was poisoned with rice tablet after about six hours; it was also able to increase systolic and diastolic blood pressure. The patient first received routine treatments, and no changes were made in the patient's clinical condition; however, after the administration of starch for the patient, better clinical conditions were observed, and he was discharged from the hospital after healing [11].

So far, gastric lavage using permanganate potassium and activated carbon in the poisoning emergency ward have been considered routine treatments, but there are some studies that reject these therapies and do not approve them [12, 13].

Hydroxyethyl starch is routinely used for many patients in ICU to recover the patient and maintain blood pressure. Along with these goals, starch is also used for other purposes such as lowering body fluids and reducing albumin from damaged endothelial cells [14, 15].

Considering Marashi et al.'s hypothesis [5] and the results of the case report [11], it seems that starch can be an appropriate candidate for treating patients with rice tablet poisoning. So far, many studies have been conducted on rice tablet poisoning, but most of these studies have been observational, and less have been few interventional studies evaluating the methods of treatment of rice tablet poisoning.

Several studies have shown that colloid serums can treat problems similar to those generated by rice tablet poisoning. For example, some researchers believe that, as compared with crystalloid serums, colloid serums remain in the vascular space for a longer time and can quickly result in hemodynamic stability [16]. In addition, another study has shown that a hydroxyethyl starch solution can reduce vascular permeability and modify vasodilatation solutions that occur in conditions such as sepsis and burns [17].

Some other studies have shown that hydroxyethyl starch improves prognosis in severe hemorrhagic shock, increases cardiac output and tissue oxygenation, and decreases blood lactate levels in cases such as septic shock [14, 18].

However, the lack of a significant difference in the present study may be due to two reasons: first, there is no significant difference between normal saline and starch in terms of increasing the blood pressure of patients with poisoning; second, there is a significant difference between the two groups in reality, but due to the small sample size in the present study, we did not observe a significant difference. In other words, the power of our study was not high enough to show a significant difference between the two groups. Therefore, it is necessary to conduct further studies with a higher sample size to confirm the results of the present study.

It should be noted that the number of survival in the two groups was equal, and there was no significant difference between the two groups in terms of the outcome of the study. However, one of the most important factors that may affect the outcome of the study is the length of time from taking the tablets until hospitalization. Proper care for patients in the early hours after taking rice tablets is the most important factor in the success of treatment for patients with this type of poisoning. Comparing the survivors and those who died, people who died were those who were hospitalized long after taking the tablet. This suggests that people who were admitted to the hospital and received treatment services earlier were more likely to survive. However, people in the starch group who died were hospitalized much longer after taking the tablet, as compared with the people in the normal saline group. We guess the high percentage of deaths in the starch group that was equal to it in the normal saline group can be due to different average time to reach the hospital between the two groups. Although this difference was not statistically significant, the absolute value of this average time was considerably higher in the starch group compared with the normal saline group (11.3 hours versus 3.14 hours). Perhaps, if the two groups were more similar in terms of this factor, the number of dead persons in the starch group was expected to be lower, and there would be a significant difference between the two groups, showing a higher level of efficiency in the starch group. It is better to consider this factor in later studies.

Finally, the results of the present study indicate that starch is as effective as normal saline in the treatment of patients with rice tablet poisoning and can be a good alternative for normal saline. However, more studies are needed to confirm this hypothesis. Further introspective studies, similar to clinical trial studies, should be conducted to confirm this finding.

## Figures and Tables

**Table 1:** Demographic characteristics of the participants.

Variable		Total number (%)	Starch group number (%)	Normal saline group number (%)	*P* value^a^
Age group	10–20	10 (28.6%)	7 (38.9%)	3 (17.6%)	0.272
	20–30	15 (42.9%)	5 (27.8%)	10 (58.8%)	
	30–40	6 (17.1%)	4 (22.2%)	2 (11.8%)	
	40–50	4 (11.4%)	2 (11.1%)	2 (11.8%)	

Sex	Male	20 (57.1%)	11 (61.1%)	9 (52.9%)	0.738
	Female	15 (42.9%)	7 (38.9%)	8 (47.1%)	

Job status	Self-employed	20 (57.1%)	10 (55.6%)	10 (58.8%)	1
	Unemployed	15 (42.9%)	8 (44.4%)	7 (41.2%)	

Education	Illiterate or under high school diploma	5 (14.3%)	2 (10.112%)	3 (17.6%)	0.208
	Diploma	28 (80%)	16 (88.9%)	12 (70.6%)	
	Higher than diploma	2 (5.7%)	0 (0%)	2 (11.8%)	

^a^Chi-square test.

**Table 2:** Comparison of systolic blood pressure in the normal saline and starch groups.

Group	Before treatment Mean (SD)	After treatment Mean (SD)	Difference	*P* value^a^
HES	72.67 (14.49)	94 (24.45)	21.33 (19)	0.001
NS	68.59 (8.3)	85.18 (19.9)	16.58 (18.75)	0.004
*P* value^b^	0.313	0.245	0.463	

HES, hydroxyethyl starch; NS, normal saline; SD, standard deviation. ^a^Paired *t-*test (or Wilcoxon test for nonnormality condition). ^b^Independent *t*-test (or Mann–Whitney *U* test for nonnormality condition).

## Data Availability

The data that support the findings of this study are available from the corresponding author, MA, upon reasonable request.
